# 6,7-Bis(methyl­sulfan­yl)-2,3-[(3,6,9-trioxaundecane-1,11-di­yl)bis­(sulfanediylmethyl­ene)]-1,4,5,8-tetra­thia­fulvalene

**DOI:** 10.1107/S160053681001192X

**Published:** 2010-04-10

**Authors:** Rui-Bin Hou, Bao Li, Bing-Zhu Yin, Li-Xin Wu

**Affiliations:** a Key Laboratory of Organism Functional Factors of the Changbai Moutain, Yanbian University, Ministry of Education, Yanji 133002, People’s Republic of China; b State Key Laboratory of Supramolecular Structure and Materials, College of Chemistry, Jilin University, Changchun 130012, People’s Republic of China

## Abstract

In the title compound, C_18_H_26_O_3_S_8_, the two five-membered rings exhibit envelope conformations. The two S atoms in the 17-membered macrocycle deviate from the plane of the fused five-membered ring by 1.429 (3) and −1.434 (3) Å in opposite directions.

## Related literature

For background to dithia­crown ether annulated tetra­thia­fulvalenes, see: Otsubo & Ogura (1985 ▶); Moore *et al.* (2000 ▶). For details of the synthesis, see: Chen *et al.* (2005 ▶). For a related structure, see Hou *et al.* (2009 ▶).
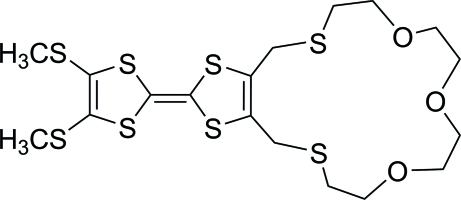


## Experimental

### 

#### Crystal data

C_18_H_26_O_3_S_8_
*M* *_r_* = 546.87
Triclinic, 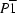

*a* = 9.715 (5) Å
*b* = 11.585 (7) Å
*c* = 12.548 (5) Å
α = 98.37 (2)°
β = 112.112 (18)°
γ = 103.94 (2)°
*V* = 1225.3 (11) Å^3^
*Z* = 2
Mo *K*α radiation
μ = 0.75 mm^−1^
*T* = 290 K
0.13 × 0.11 × 0.11 mm

#### Data collection

Rigaku R-AXIS RAPID diffractometer
Absorption correction: multi-scan (*ABSCOR*; Higashi, 1995 ▶) *T*_min_ = 0.909, *T*_max_ = 0.922
12067 measured reflections
5535 independent reflections
4519 reflections with *I* > 2σ(*I*)
*R*_int_ = 0.021

#### Refinement

*R*[*F* ^2^ > 2σ(*F* ^2^)] = 0.045
*wR*(*F* ^2^) = 0.125
*S* = 1.09
5535 reflections
264 parameters
H-atom parameters constrained
Δρ_max_ = 1.19 e Å^−3^
Δρ_min_ = −0.84 e Å^−3^

### 

Data collection: *RAPID-AUTO* (Rigaku, 1998 ▶); cell refinement: *RAPID-AUTO*; data reduction: *CrystalStructure* (Rigaku/MSC and Rigaku, 2002 ▶); program(s) used to solve structure: *SHELXS97* (Sheldrick, 2008 ▶); program(s) used to refine structure: *SHELXL97* (Sheldrick, 2008 ▶); molecular graphics: *PLATON* (Spek, 2009 ▶); software used to prepare material for publication: *SHELXL97*.

## Supplementary Material

Crystal structure: contains datablocks global, I. DOI: 10.1107/S160053681001192X/cv2706sup1.cif

Structure factors: contains datablocks I. DOI: 10.1107/S160053681001192X/cv2706Isup2.hkl

Additional supplementary materials:  crystallographic information; 3D view; checkCIF report

## Figures and Tables

**Fig. 1.**
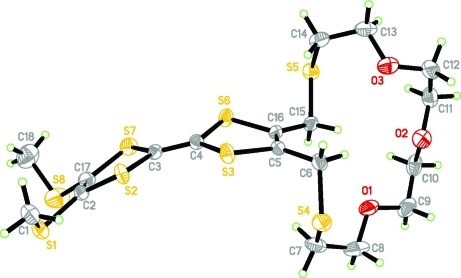
The molecular structure of (I) showing the atom numbering and 30% probability displacement ellipsoids.

## supplementary crystallographic information

### Comment

Dithiacrown ether annulated tetrathiafulvalenes (TTF) have been received great attention as sensor molecules for various metal cations (Otsubo & Ogura, 1985; Moore *et al.*, 2000). These sensors can recognize selectively the various metal cations to signal electrochemical information. We fused TTF unit with a extended dithiacrown ether to synthesize the title compound, (I). Herewith we report its crystal structure.

In  (I) (Fig. 1), all bond lengths and angles are normal and comparable to those observed in the related structure (Hou *et al.*, 2009). Two five-membered rings have an envelope conformation. Two S atoms in the 17-membered macrocycle deviate from the plane of the fused five-membered ring in opposite directions at 1.429 (3) and -1.434 (3) Å, respectively. The mean planes of two five-membered rings form a dihedral angle of 29.84 (10) °.

### Experimental

The title compound was prepared according to the literature (Chen *et al.*, 2005). Single crystals suitable for X-ray diffraction were prepared by slow evaporation a mixture of dichloromethane and petroleum (60-90 °C) at room temperature.

### Refinement

Carbon-bound H-atoms were placed in calculated positions (C—H 0.96 and 0.97 Å) and were included in the refinement in the riding model approximation, with U_iso_(H) = 1.5 or 1.2 U_eq_(C).

### Crystal data

**Table:**

C_18_H_26_O_3_S_8_	*Z* = 2
*M**_r_* = 546.87	*F*(000) = 572
Triclinic, *P*1	*D*_x_ = 1.482 Mg m^−^^3^
Hall symbol: -P 1	Mo *K*α radiation, λ = 0.71073 Å
*a* = 9.715 (5) Å	Cell parameters from 8201 reflections
*b* = 11.585 (7) Å	θ = 3.1–27.5°
*c* = 12.548 (5) Å	µ = 0.75 mm^−^^1^
α = 98.37 (2)°	*T* = 290 K
β = 112.112 (18)°	Block, yellow
γ = 103.94 (2)°	0.13 × 0.11 × 0.11 mm
*V* = 1225.3 (11) Å^3^	

### Data collection

**Table:**

Rigaku R-AXIS RAPID diffractometer	5535 independent reflections
Radiation source: fine-focus sealed tube	4519 reflections with *I* > 2σ(*I*)
graphite	*R*_int_ = 0.021
ω scans	θ_max_ = 27.5°, θ_min_ = 3.2°
Absorption correction: multi-scan (*ABSCOR*; Higashi, 1995)	*h* = −12→11
*T*_min_ = 0.909, *T*_max_ = 0.922	*k* = −14→15
12067 measured reflections	*l* = −15→16

### Refinement

**Table:**

Refinement on *F*^2^	Primary atom site location: structure-invariant direct methods
Least-squares matrix: full	Secondary atom site location: difference Fourier map
*R*[*F*^2^ > 2σ(*F*^2^)] = 0.045	Hydrogen site location: inferred from neighbouring sites
*wR*(*F*^2^) = 0.125	H-atom parameters constrained
*S* = 1.09	*w* = 1/[σ^2^(*F*_o_^2^) + (0.0606*P*)^2^ + 0.6546*P*] where *P* = (*F*_o_^2^ + 2*F*_c_^2^)/3
5535 reflections	(Δ/σ)_max_ = 0.004
264 parameters	Δρ_max_ = 1.19 e Å^−^^3^
0 restraints	Δρ_min_ = −0.84 e Å^−^^3^

### Special details

**Table:**

Experimental. (See detailed section in the paper)
Geometry. All esds (except the esd in the dihedral angle between two l.s. planes) are estimated using the full covariance matrix. The cell esds are taken into account individually in the estimation of esds in distances, angles and torsion angles; correlations between esds in cell parameters are only used when they are defined by crystal symmetry. An approximate (isotropic) treatment of cell esds is used for estimating esds involving l.s. planes.
Refinement. Refinement of *F*^2^ against ALL reflections. The weighted *R*-factor wR and goodness of fit *S* are based on *F*^2^, conventional *R*-factors *R* are based on *F*, with *F* set to zero for negative *F*^2^. The threshold expression of *F*^2^ > σ(*F*^2^) is used only for calculating *R*-factors(gt) etc. and is not relevant to the choice of reflections for refinement. *R*-factors based on *F*^2^ are statistically about twice as large as those based on *F*, and *R*- factors based on ALL data will be even larger.

### Fractional atomic coordinates and isotropic or equivalent isotropic displacement parameters (Å^2^)

**Table:**

	*x*	*y*	*z*	*U*_iso_*/*U*_eq_	
C1	0.8329 (4)	0.2619 (3)	−0.0795 (3)	0.0673 (8)	
H1A	0.9100	0.3281	−0.0122	0.101*	
H1B	0.8797	0.2016	−0.0963	0.101*	
H1C	0.7472	0.2243	−0.0620	0.101*	
C2	0.6741 (3)	0.4211 (2)	−0.1600 (2)	0.0461 (6)	
C3	0.6468 (3)	0.6002 (2)	−0.0284 (2)	0.0378 (5)	
C4	0.6879 (3)	0.7034 (2)	0.0568 (2)	0.0375 (5)	
C5	0.8608 (3)	0.9048 (2)	0.2248 (2)	0.0377 (5)	
C6	1.0049 (3)	0.9939 (2)	0.3263 (2)	0.0453 (6)	
H6A	1.0222	0.9641	0.3970	0.054*	
H6B	0.9875	1.0724	0.3417	0.054*	
C7	1.1338 (4)	1.0744 (4)	0.1696 (3)	0.0672 (9)	
H7A	1.1946	1.0522	0.1287	0.081*	
H7B	1.0238	1.0324	0.1167	0.081*	
C8	1.1631 (4)	1.2111 (4)	0.1912 (4)	0.0809 (11)	
H8A	1.2668	1.2551	0.2548	0.097*	
H8B	1.1577	1.2362	0.1197	0.097*	
C9	1.0704 (4)	1.3669 (3)	0.2552 (3)	0.0717 (9)	
H9A	1.0745	1.4025	0.1906	0.086*	
H9B	1.1688	1.4080	0.3253	0.086*	
C10	0.9373 (4)	1.3834 (3)	0.2801 (3)	0.0631 (8)	
H10A	0.9445	1.4696	0.2948	0.076*	
H10B	0.8383	1.3365	0.2124	0.076*	
C11	0.8268 (4)	1.3533 (3)	0.4170 (3)	0.0631 (8)	
H11A	0.7245	1.3175	0.3497	0.076*	
H11B	0.8427	1.4396	0.4478	0.076*	
C12	0.8366 (4)	1.2872 (3)	0.5112 (3)	0.0684 (9)	
H12A	0.9438	1.3157	0.5724	0.082*	
H12B	0.7701	1.3060	0.5479	0.082*	
C13	0.6293 (4)	1.0953 (3)	0.4346 (3)	0.0646 (8)	
H13A	0.5700	1.1524	0.4205	0.077*	
H13B	0.6215	1.0642	0.5006	0.077*	
C14	0.5624 (4)	0.9902 (3)	0.3249 (3)	0.0579 (7)	
H14A	0.4659	0.9349	0.3191	0.070*	
H14B	0.6358	0.9445	0.3335	0.070*	
C15	0.7020 (3)	1.0495 (2)	0.1709 (2)	0.0424 (5)	
H15A	0.6948	1.0792	0.1011	0.051*	
H15B	0.7895	1.1095	0.2398	0.051*	
C16	0.7328 (3)	0.9291 (2)	0.1574 (2)	0.0369 (5)	
C17	0.5549 (3)	0.4512 (3)	−0.2335 (2)	0.0542 (7)	
C18	0.2899 (5)	0.2958 (4)	−0.4241 (4)	0.0993 (14)	
H18A	0.3042	0.2321	−0.3839	0.149*	
H18B	0.2354	0.2598	−0.5086	0.149*	
H18C	0.2296	0.3380	−0.3983	0.149*	
O1	1.0472 (3)	1.2390 (2)	0.2231 (2)	0.0649 (6)	
O2	0.9459 (2)	1.3412 (2)	0.38150 (17)	0.0585 (5)	
O3	0.7891 (2)	1.1573 (2)	0.46389 (19)	0.0605 (5)	
S1	0.76152 (11)	0.32107 (7)	−0.20652 (7)	0.0659 (2)	
S2	0.75246 (8)	0.49614 (6)	−0.00808 (5)	0.04786 (17)	
S3	0.85994 (8)	0.75086 (6)	0.19036 (5)	0.04658 (17)	
S4	1.18116 (8)	1.01831 (8)	0.30089 (7)	0.0583 (2)	
S5	0.52206 (8)	1.03736 (6)	0.18849 (6)	0.04905 (17)	
S6	0.57706 (7)	0.80320 (6)	0.04603 (6)	0.04656 (17)	
S7	0.48777 (8)	0.55840 (7)	−0.16902 (6)	0.05239 (18)	
S8	0.47241 (13)	0.40014 (12)	−0.39012 (7)	0.0970 (4)	

### Atomic displacement parameters (Å^2^)

**Table:**

	*U*^11^	*U*^22^	*U*^33^	*U*^12^	*U*^13^	*U*^23^
C1	0.070 (2)	0.0609 (19)	0.077 (2)	0.0254 (16)	0.0397 (18)	0.0044 (16)
C2	0.0520 (15)	0.0406 (13)	0.0391 (12)	−0.0002 (11)	0.0261 (11)	−0.0012 (10)
C3	0.0382 (12)	0.0360 (12)	0.0364 (11)	0.0085 (9)	0.0153 (9)	0.0096 (9)
C4	0.0378 (12)	0.0352 (11)	0.0389 (11)	0.0118 (9)	0.0159 (9)	0.0096 (9)
C5	0.0369 (12)	0.0373 (12)	0.0380 (11)	0.0115 (9)	0.0178 (10)	0.0038 (9)
C6	0.0406 (13)	0.0497 (14)	0.0390 (12)	0.0129 (11)	0.0150 (10)	0.0013 (10)
C7	0.0522 (17)	0.100 (3)	0.0579 (17)	0.0260 (17)	0.0347 (15)	0.0127 (17)
C8	0.059 (2)	0.110 (3)	0.091 (3)	0.019 (2)	0.0487 (19)	0.045 (2)
C9	0.078 (2)	0.0544 (18)	0.069 (2)	−0.0044 (16)	0.0316 (17)	0.0209 (15)
C10	0.081 (2)	0.0383 (14)	0.0566 (16)	0.0106 (14)	0.0199 (15)	0.0147 (12)
C11	0.0613 (19)	0.0534 (17)	0.0645 (18)	0.0204 (15)	0.0203 (15)	0.0021 (14)
C12	0.064 (2)	0.081 (2)	0.0460 (15)	0.0117 (17)	0.0222 (14)	−0.0003 (14)
C13	0.0501 (16)	0.091 (2)	0.0611 (18)	0.0179 (16)	0.0329 (14)	0.0263 (17)
C14	0.0535 (17)	0.0573 (17)	0.078 (2)	0.0194 (14)	0.0388 (15)	0.0305 (15)
C15	0.0411 (13)	0.0337 (12)	0.0526 (14)	0.0112 (10)	0.0212 (11)	0.0106 (10)
C16	0.0335 (11)	0.0338 (11)	0.0437 (12)	0.0084 (9)	0.0193 (10)	0.0076 (9)
C17	0.0500 (15)	0.0618 (17)	0.0353 (12)	−0.0017 (13)	0.0199 (12)	−0.0028 (11)
C18	0.090 (3)	0.098 (3)	0.070 (2)	0.000 (2)	0.016 (2)	0.008 (2)
O1	0.0606 (13)	0.0569 (12)	0.0870 (15)	0.0080 (10)	0.0468 (12)	0.0237 (11)
O2	0.0605 (12)	0.0613 (12)	0.0532 (11)	0.0206 (10)	0.0208 (10)	0.0212 (9)
O3	0.0486 (11)	0.0760 (14)	0.0617 (12)	0.0191 (10)	0.0265 (10)	0.0257 (11)
S1	0.0891 (6)	0.0546 (4)	0.0623 (4)	0.0167 (4)	0.0508 (4)	−0.0005 (3)
S2	0.0601 (4)	0.0414 (3)	0.0366 (3)	0.0208 (3)	0.0156 (3)	0.0012 (2)
S3	0.0475 (4)	0.0435 (3)	0.0412 (3)	0.0221 (3)	0.0095 (3)	0.0030 (2)
S4	0.0325 (3)	0.0715 (5)	0.0582 (4)	0.0171 (3)	0.0114 (3)	0.0018 (3)
S5	0.0395 (3)	0.0518 (4)	0.0557 (4)	0.0219 (3)	0.0173 (3)	0.0103 (3)
S6	0.0351 (3)	0.0367 (3)	0.0556 (4)	0.0113 (2)	0.0094 (3)	0.0050 (3)
S7	0.0433 (4)	0.0610 (4)	0.0404 (3)	0.0105 (3)	0.0102 (3)	0.0091 (3)
S8	0.0877 (7)	0.1331 (10)	0.0378 (4)	−0.0061 (6)	0.0267 (4)	−0.0001 (5)

### Geometric parameters (Å, °)

**Table:**

C1—S1	1.790 (4)	C10—O2	1.408 (3)
C1—H1A	0.9600	C10—H10A	0.9700
C1—H1B	0.9600	C10—H10B	0.9700
C1—H1C	0.9600	C11—O2	1.415 (4)
C2—C17	1.341 (4)	C11—C12	1.485 (5)
C2—S1	1.748 (3)	C11—H11A	0.9700
C2—S2	1.753 (3)	C11—H11B	0.9700
C3—C4	1.345 (3)	C12—O3	1.425 (4)
C3—S2	1.750 (3)	C12—H12A	0.9700
C3—S7	1.753 (2)	C12—H12B	0.9700
C4—S6	1.746 (2)	C13—O3	1.420 (4)
C4—S3	1.756 (2)	C13—C14	1.503 (5)
C5—C16	1.337 (3)	C13—H13A	0.9700
C5—C6	1.492 (3)	C13—H13B	0.9700
C5—S3	1.770 (3)	C14—S5	1.803 (3)
C6—S4	1.821 (3)	C14—H14A	0.9700
C6—H6A	0.9700	C14—H14B	0.9700
C6—H6B	0.9700	C15—C16	1.496 (3)
C7—C8	1.504 (5)	C15—S5	1.818 (3)
C7—S4	1.798 (4)	C15—H15A	0.9700
C7—H7A	0.9700	C15—H15B	0.9700
C7—H7B	0.9700	C16—S6	1.767 (3)
C8—O1	1.419 (4)	C17—S8	1.761 (3)
C8—H8A	0.9700	C17—S7	1.765 (3)
C8—H8B	0.9700	C18—S8	1.742 (5)
C9—O1	1.416 (4)	C18—H18A	0.9600
C9—C10	1.487 (5)	C18—H18B	0.9600
C9—H9A	0.9700	C18—H18C	0.9600
C9—H9B	0.9700		
			
S1—C1—H1A	109.5	O2—C11—H11B	110.3
S1—C1—H1B	109.5	C12—C11—H11B	110.3
H1A—C1—H1B	109.5	H11A—C11—H11B	108.6
S1—C1—H1C	109.5	O3—C12—C11	111.5 (2)
H1A—C1—H1C	109.5	O3—C12—H12A	109.3
H1B—C1—H1C	109.5	C11—C12—H12A	109.3
C17—C2—S1	124.5 (2)	O3—C12—H12B	109.3
C17—C2—S2	116.3 (2)	C11—C12—H12B	109.3
S1—C2—S2	119.01 (17)	H12A—C12—H12B	108.0
C4—C3—S2	121.99 (19)	O3—C13—C14	109.6 (2)
C4—C3—S7	124.10 (19)	O3—C13—H13A	109.7
S2—C3—S7	113.89 (13)	C14—C13—H13A	109.7
C3—C4—S6	123.71 (19)	O3—C13—H13B	109.7
C3—C4—S3	122.82 (19)	C14—C13—H13B	109.7
S6—C4—S3	113.46 (13)	H13A—C13—H13B	108.2
C16—C5—C6	126.9 (2)	C13—C14—S5	114.0 (2)
C16—C5—S3	116.77 (18)	C13—C14—H14A	108.8
C6—C5—S3	116.37 (18)	S5—C14—H14A	108.8
C5—C6—S4	114.01 (17)	C13—C14—H14B	108.8
C5—C6—H6A	108.7	S5—C14—H14B	108.8
S4—C6—H6A	108.7	H14A—C14—H14B	107.7
C5—C6—H6B	108.7	C16—C15—S5	112.39 (16)
S4—C6—H6B	108.7	C16—C15—H15A	109.1
H6A—C6—H6B	107.6	S5—C15—H15A	109.1
C8—C7—S4	115.4 (2)	C16—C15—H15B	109.1
C8—C7—H7A	108.4	S5—C15—H15B	109.1
S4—C7—H7A	108.4	H15A—C15—H15B	107.9
C8—C7—H7B	108.4	C5—C16—C15	127.2 (2)
S4—C7—H7B	108.4	C5—C16—S6	116.68 (18)
H7A—C7—H7B	107.5	C15—C16—S6	115.94 (18)
O1—C8—C7	108.5 (3)	C2—C17—S8	124.0 (2)
O1—C8—H8A	110.0	C2—C17—S7	117.57 (19)
C7—C8—H8A	110.0	S8—C17—S7	118.18 (19)
O1—C8—H8B	110.0	S8—C18—H18A	109.5
C7—C8—H8B	110.0	S8—C18—H18B	109.5
H8A—C8—H8B	108.4	H18A—C18—H18B	109.5
O1—C9—C10	108.2 (2)	S8—C18—H18C	109.5
O1—C9—H9A	110.1	H18A—C18—H18C	109.5
C10—C9—H9A	110.1	H18B—C18—H18C	109.5
O1—C9—H9B	110.1	C9—O1—C8	113.5 (3)
C10—C9—H9B	110.1	C10—O2—C11	114.0 (2)
H9A—C9—H9B	108.4	C13—O3—C12	113.9 (3)
O2—C10—C9	107.8 (3)	C2—S1—C1	101.77 (14)
O2—C10—H10A	110.2	C3—S2—C2	95.28 (13)
C9—C10—H10A	110.2	C4—S3—C5	94.67 (11)
O2—C10—H10B	110.2	C7—S4—C6	103.95 (13)
C9—C10—H10B	110.2	C14—S5—C15	102.32 (13)
H10A—C10—H10B	108.5	C4—S6—C16	94.86 (12)
O2—C11—C12	107.1 (3)	C3—S7—C17	94.12 (13)
O2—C11—H11A	110.3	C18—S8—C17	102.48 (18)
C12—C11—H11A	110.3		
